# The need for patient-focused therapy for children and teenagers with allergic rhinitis: a case-based review of current European practice

**DOI:** 10.1186/s13601-014-0044-5

**Published:** 2015-01-24

**Authors:** Alexandra F Santos, Luis Miguel Borrego, Giuseppina Rotiroti, Glenis Scadding, Graham Roberts

**Affiliations:** Department of Paediatric Allergy, Division of Asthma, Allergy & Lung Biology, King’s College London, London, UK; MRC & Asthma UK Centre in Allergic Mechanisms of Asthma, London, UK; Immunoallergology Department, Coimbra University Hospital, Coimbra, Portugal; CUF Descobertas Hospital, Lisbon, Portugal; CEDOC, Nova Medical School, Universidade Nova de Lisboa, Lisbon, Portugal; The Royal National Throat, Nose and Ear Hospital & University College London Hospitals, London, UK; David Hide Asthma and Allergy Research Centre, St Mary’s Hospital, Isle of Wight, UK; Human Development and Health and Clinical Experimental Sciences Academic Subunits, University of Southampton Faculty of Medicine, Southampton, UK; Respiratory Biomedical Research Unit, University Hospital Southampton NHS Foundation Trust, Southampton, UK; Paediatric Allergy and Respiratory Medicine, University Child Health (MP803), University Hospital Southampton NHS Foundation Trust, Southampton, UK

**Keywords:** Allergy, Rhinitis, Pediatric rhinitis, Immunotherapy, Guidelines

## Abstract

Allergic rhinitis is a common problem in childhood and adolescence, with a negative impact on the quality of life of patients and their families. The treatment modalities for allergic rhinitis include allergen avoidance, anti-inflammatory symptomatic treatment and allergen specific immunotherapy. In this review, four cases of children with allergic rhinitis are presented to illustrate how the recently published EAACI Guidelines on Pediatric Allergic Rhinitis can be implemented in clinical practice.

## Introduction

**Figure 1:**
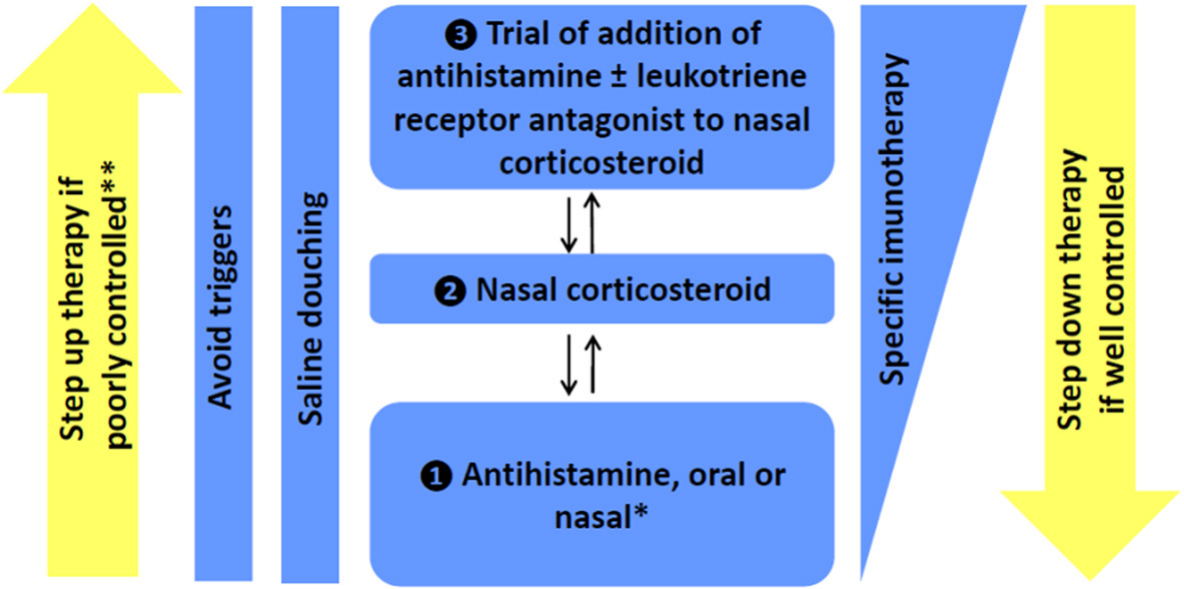
**Treatment of allergic rhinitis (7).** The entry points into therapeutic approach depend on the severity of the rhinitis symptoms. Therapy can be step up or step down depending on control obtained in response to the treatment. If less than 2 years of age and do not respond to antihistamine within a week, the diagnosis should be reconsidered before stepping up therapy. *Oral antihistamines may be better tolerated, whilst intranasal antihistamines have a more rapid onset of action. **Reconsider diagnosis if not controlled within 1-2 weeks.

Allergic rhinitis is a common problem in childhood and adolescence [1]. This is partly the reason why it is often under perceived by patients and families, under diagnosed and its impact underestimated. Allergic rhinitis causes chronic disturbing symptoms which have a negative effect on physical, social and psychological well-being, as well as on school performance of children and teenagers [2-4]. There are multiple associated co-morbidities [5], which further contribute to the direct and indirect costs of rhinitis [6]. Recently, a European Academy of Allergy and Clinical Immunology (EAACI) position paper on pediatric rhinitis was published to address the need for guidance on the management of this condition in the pediatric age group [7]. The main treatment modalities for pediatric allergic rhinitis include: avoidance of the relevant allergens, symptomatic treatment with H1-anti-histamines, intranasal corticosteroids and oral leukotriene-receptor antagonists, and allergen-specific immunotherapy (Figure 1). In this review article, we have used four pediatric cases to illustrate key aspects of the treatment of pediatric allergic rhinitis as an exercise to help implementing the aforementioned EAACI guidelines in clinical practice.

### Case 1

#### The importance of patient education and of a good nasal spray technique

Six-year-old girl presented to clinic in June with troublesome hay fever symptoms. She had significant nasal obstruction and pruritus, sneezing and watery nasal discharge. The symptoms had started in early April and were progressively worsening. In previous years, she had had similar symptoms from April to July, but was asymptomatic during the rest of the year. Her doctor had prescribed oral cetirizine and intranasal mometasone furoate about 4 weeks after the symptoms started, but this treatment did not result in significant improvement. She stopped using the intranasal corticosteroid two weeks later because she was having frequent nose bleeds and had developed nasal crusting that she associated with the use of the nasal spray.

Skin prick testing was positive to grass pollens and negative to other common airborne allergens. After checking the patient’s nasal spray technique, the allergy clinic team realised that she was not using it properly and spent some time providing appropriate training.

The patient was prescribed a very short course of rescue decongestant to open up her congested nasal airway and intranasal isotonic saline to minimise the formation of nasal crusting. She was advised to use daily intranasal mometasone furoate and cetirizine until the end of the pollen season, whilst attempting to minimise direct exposure to grass pollen. Her family was educated to start cetirizine and intranasal corticosteroids about 2 weeks before the beginning of the grass pollen season in the following years.

**Figure 2:**
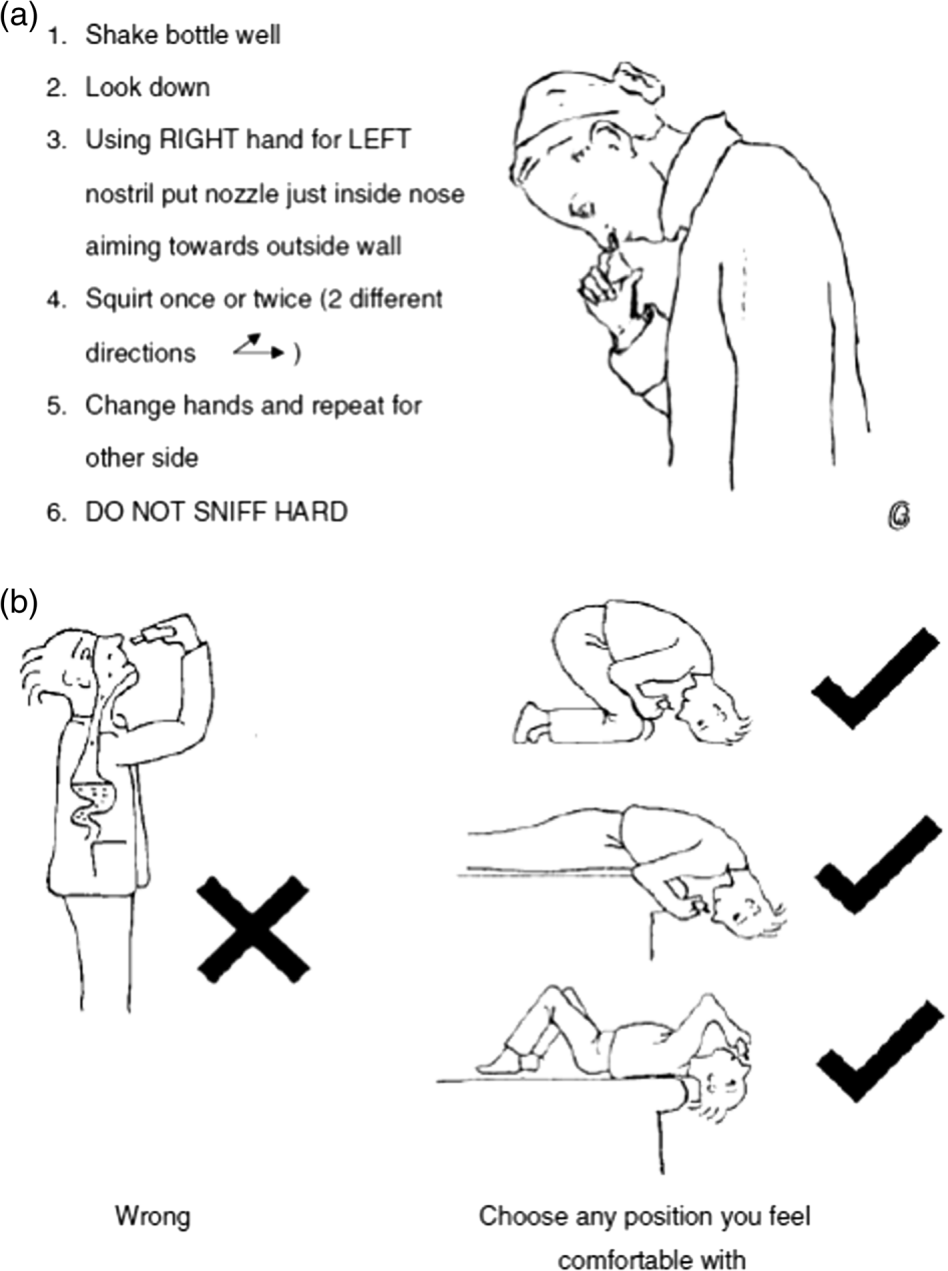
**The use of an appropriate technique for (a) application of nasal spray and (b) installation of nasal drops (8) is key for the success of the treatment.**

Case 1 highlights the importance of adherence to treatment and of a correct application technique of the nasal spray for maximum effect and for minimising side effects. Nasal drops and nasal sprays require different techniques (Figure 2) [8]. Poor technique is a common cause of treatment failure, so it is important to spend time in clinic explaining the appropriate use of these devices and providing hands-on training [9]. It is also important to explain the nature of the treatment, its safety profile and possible side effects. Patients should be given realistic expectations about the results of the treatment and should be informed that complete resolution does not usually occur in the treatment of chronic conditions such as allergic rhinitis. For example, in the case of intranasal corticosteroids, patients should be informed that they take a few days before any effects can be noticed. Appropriate information helps in ensuring concordance with therapy, which is critical for a good control of the nasal inflammation and for the improvement of the symptoms.

Minimising allergen exposure is also an important part of the management of this condition. Given her grass pollen allergy, she was advised to minimize early morning and evening activities outdoors, to avoid going out after thunderstorms or on windy days during the pollen season, to wear sunglasses when outside, to avoid mowing the grass or being near it when it is being mowed, to keep windows closed as much as possible and use air-conditioning and to wash her hair at the end of the day when she arrives home as well as to bathe her eyes and douche her nose frequently during the grass pollen season.

Finally, pollen levels rise slowly at the start of the pollen season with symptoms only presenting at a threshold level. Prior to this, exposure to small amounts of allergen will attract inflammatory cells into the nasal airway exacerbating symptoms when pollen levels rise further. Commencing hayfever treatment a few weeks prior to the expected start of the seasons, can be very helpful in delaying symptom onset and in achieving symptom control.

### Case 2

#### The role of anti-leukotrienes in the treatment of allergic rhinitis

An 8-year-old boy was referred to the Allergy clinic due to nasal symptoms consisting of rhinorrhoea, sneezing and nasal itching since he was 5. His parents reported that the symptoms usually persisted throughout the year, worsened during the winter, particularly with exposure to house dust, but had not disturbed his sleep or daily activities. While exercising, he usually developed wheeze and cough that subsided with rest. His physical examination was normal and skin prick tests were positive for house dust mite (HDM). Allergen avoidance and a once daily, non-sedative H1-antihistamine (desloratadine) resulted in improvement of the nasal symptoms but his exercise-induced complaints persisted. An anti-leukotriene (montelukast) was therefore added resulting in better control of the lower respiratory symptoms.

This patient had classic symptoms and signs of allergic rhinitis with rhinorrhoea, pruritus and sneezing. The presence of allergic sensitization to HDM suggests a diagnosis of allergic rhinitis.

According to the Allergic Rhinitis and its Impact on Asthma (ARIA) guidelines [10], his rhinitis would be classified as persistent based on the duration of symptoms and mild according to the impact of the disease [10]. He also had wheeze in association with exercise. Various studies have shown that rhinitis and bronchial hyper-reactivity frequently co-exist in children [11-14] and that treatment of rhinitis can improve asthma control [15].

Allergen avoidance measures and anti-histamine therapy led to partial clinical improvement. It has been reported that allergen avoidance should be the first intervention for allergic rhinitis although interventional studies suggest that it is of limited value for allergens such as house dust mite. Regarding therapy, antihistamines can be used as first-line therapy, although nasal corticosteroids are more effective particularly in reducing mucosal oedema and may also have beneficial effects on asthma [13,15-19]. Nevertheless, antihistamines may be preferred in mild rhinitis, especially if there is no nasal obstruction, as in this case. Adding montelukast to the treatment improved symptom control. Anti-leukotriene receptor antagonists have been shown to be effective for controlling exercise-induced asthma and preventing the seasonal decrease in lung function parameters in patients with grass pollen allergy [20]. They may be used as add-on therapy to control rhinitis in patients with concomitant asthma [13,16-19].

### Case 3

#### The treatment of coexisting conjunctivitis in allergic rhinitis

A 10 year-old girl was referred to the Allergy outpatient clinic for rhino-conjunctivitis of one year’s duration. Her symptoms of rhinorrhoea, nasal blockage, sneezing and itching started when she was 7. She reported that her symptoms only occurred from March to June. They had now worsened with the addition of bilateral ocular *pruritus*, conjunctival *hyperaemia* and watering eyes. During these episodes she could not do her usual daily activities outdoors and did not sleep well. Physical examination was normal and skin prick tests were positive for grass pollen.

Considerable improvement occurred with allergen avoidance and oral levocetirizine once daily. After adding a nasal corticosteroid (mometasone furoate) once a day to the treatment, complete clinical remission occurred with good control of both ocular and nasal symptoms.

In the presence of rhinorrhoea, nasal obstruction, pruritus and sneezing [21] which develop seasonally and sensitization to pollen [10], a diagnosis of allergic rhinitis was made. According to the ARIA guidelines [10], it was classified as persistent rhinitis, since symptoms occurred more than 4 days a week and more than 4 weeks a year and as moderate/severe, since it affected her daily activities and sleep [10]. This case highlights that despite having only seasonal symptoms, patients can present with persistent moderate-severe rhinitis, which has implications for therapy.

This child also had allergic conjunctivitis, with bilateral ocular pruritus, hyperaemia and watery eyes, which is very common in patients with pollen-induced allergic rhinitis. Allergic conjunctivitis is the commonest co-morbidity associated with allergic rhinitis [22].

As described before, minimization of contact with the relevant allergens should be the first-line intervention. So, she was advised to avoid outdoor activities during the periods of high pollen count, to wear sunglasses when outside and to open the windows only during the evening and night.

Nasal corticosteroids were prescribed, ameliorating nasal and ocular symptoms. Nasal corticosteroids are considered to be the most effective treatment to control nasal inflammation in moderate to severe allergic rhinitis [13,16-18], especially in the presence of nasal obstruction [7,18]. Moreover, some studies have shown that fluticasone furoate and mometasone furoate improve conjunctivitis since some of the eye symptoms result from nasal inflammation and nasal-ocular reflex activation [23]. A recent systematic review and meta-analysis suggest that leukotriene receptor antagonists may also have a beneficial effect in ocular symptoms of seasonal allergic rhinitis [24].

Nasal corticosteroids are well tolerated, being recommended for children and adolescents with allergic rhinitis, from the age of two [23,25-27]. Newer nasal corticosteroids (e.g. fluticasone propionate [28], mometasone furoate [29]) are safe, and do not impair growth velocity [30], as opposed to older nasal corticosteroids, such as beclomethasone and budesonide. However, a recent study shows small impairment of growth with fluticasone furoate administered over a one-year period to prepubescent children with perennial allergic rhinitis [31]. Therefore, when prescribing nasal corticosteroids to children, one should balance the benefits and risks, prefer the ones with documented fewer adverse effects and explain to parents the safety and efficacy of this treatment in order to avoid loss of adherence to therapy and “steroid-phobia”.

Finally, non-sedative antihistamines are useful as add-on therapy to nasal corticosteroids [7,10,16-18,32] and were prescribed for this patient with good results. First-generation antihistamines are not recommended for the treatment of allergic rhinitis as they cause sedation and may affect cognitive function and reduce academic and/or work performance [2,33].

### Case 4

#### The role of allergen specific immunotherapy in the treatment of allergic rhinitis in children

Twelve-year-old boy presented with very disruptive symptoms of rhinitis with significant nasal obstruction as well as sneezing, rhinorrhoea and very disturbing nasal and ocular pruritus. These symptoms developed every year during the summer months and were persistent and severe, affecting his ability to sleep and his performance at school. His exam marks were lower in his summer examinations compared to those earlier in the year. He also felt that his nose problems were restricting his sport and social activities during the period when the weather was good; he liked playing outside. He was tested for different airborne allergens and both skin and specific IgE testing showed sensitization to grass pollen confirming grass pollen allergy. The previous years, he had been prescribed loratadine, intranasal mometasone furoate, montelukast and sodium cromoglicate eye drops, which he was taking. Despite good adherence, he continued to have poor disease control.

Given his continued symptoms, that were impacting on this quality of life, despite optimal pharmacotherapy, he was started on sublingual immunotherapy to grass pollen. He took the first dose in clinic and continued with the treatment at home. He initially had some local pruritus but this settled after a couple of weeks. One year into this treatment, he was already feeling some improvement and was able to reduce the medication he was taking to loratadine only.

Allergen-specific immunotherapy (IT) is the only disease-modifying treatment for allergic rhinitis. It is able to change the natural history of this condition and to provide long-term remission [34,35]. It is indicated in patients over 5 years old with demonstrable IgE to clinically relevant allergens, particularly in patients where pharmacological treatment has failed to control symptoms [36]. Since he was having troublesome symptoms despite maximum pharmacological therapy and the symptoms were due to grass pollen exposure to which he had detectable IgE, he was a good candidate for this treatment. There are standardized extracts to grass pollen commercially available to administer via the subcutaneous or the sublingual route [37,38]. Although there are very few head-to-head studies comparing subcutaneous immunotherapy (SCIT) and sublingual immunotherapy (SLIT), both forms are effective if appropriately used [39,40]. In terms of safety, SCIT more frequently causes systemic adverse events while SLIT tends to cause more local side effects, which are usually mild and resolve with continuation of treatment [41,42]. Severe adverse events are commonly associated with uncontrolled asthma, high allergen exposure during therapy, concomitant diseases such as severe infections and inexperienced health care staff. Premedication with an antihistamine may decrease the rate of adverse effects [43]. The decision on whether to do SLIT or SCIT depends on a variety of factors, including patient’s preference about home-based versus hospital-based treatment, fear of injections, costs and concordance.[44]. In children, SLIT is more widely accepted but may have lower patient adherence [45]. Although SLIT is given at home, the first dose should be given at the doctor’s office. This is also the opportunity to give detailed instructions about how to administer the treatment and about the precautions to be taken. Patients should be informed about possible adverse reactions and about the ways to treat them. Apart from the effectiveness in reducing symptoms and medication use, another potential advantage of allergen-specific immunotherapy is its preventative effects in reducing asthma and the development of further allergic sensitizations [46-50]. This is particularly important in the pediatric age groups. When clinically indicated, IT should be started early in the disease process, before significant remodelling and fixed airway obstruction has developed in the case of patients with asthma. As allergen-specific immunotherapy is the only disease-modifying treatment available for allergic rhinitis and respiratory allergy, it may be considered as a therapeutic option even before trying maximal therapy, depending on individual cases, clinical practice and finance.

## Conclusions

1. Patient education and appropriate nasal device training are very important for an effective and safe treatment of allergic rhinitis in children.
2. Allergen avoidance is part of the treatment of allergic rhinitis.
3. Symptomatic relief and reduction of nasal inflammation may be obtained with nasal corticosteroids, which are globally the most effective therapy. Oral anti-histamines and anti-leukotrienes can also prove effective.
4. Allergen-specific immunotherapy is the only disease-modifying treatment for allergic rhinitis and has the potential to prevent the development of further allergic sensitization and asthma.

## Abbreviations

ARIA: Allergic rhinitis and its impact on asthma
EAACI: European Academy of Allergy and Clinical Immunology
HDM: House dust mite
IT: Immunotherapy
SCIT: Subcutaneous immunotherapy
SLIT: Sublingual immunotherapy
